# Reference Intervals for Biochemical Analytes in Clinically Healthy Adult Lusitano Horses

**DOI:** 10.3390/vetsci12070656

**Published:** 2025-07-11

**Authors:** Maria João Pires, Mário Cotovio, Felisbina Queiroga, Carlos André Pires, Ana C. Silvestre-Ferreira

**Affiliations:** 1 Veterinary Sciences Department, University of Trás-os-Montes and Alto Douro, 5000-801 Vila Real, Portugal; joaomp@utad.pt (M.J.P.); fqueirog@utad.pt (F.Q.); 2 CITAB, Centre for Research and Technology of Agro-Environment and Biological Sciences, University of Trás-os-Montes and Alto Douro, 5000-801 Vila Real, Portugal; 3 Faculty of Veterinary Medicine, Lusófona University, Campo Grande 376, 1749-024 Lisbon, Portugal; p4980@ulusofona.pt; 4 CECAV-Veterinary and Animal Research Center, University of Trás-os-Montes and Alto Douro, 5000-801 Vila Real, Portugal; 5 Associate Laboratory for Animal and Veterinary Sciences (AL4AnimalS), 5000-801 Vila Real, Portugal; 6 Center for Research in Neuropsychology and Cognitive and Behavioral Intervention (CINEICC), University of Coimbra, Largo D. Dinis, 3004-531 Coimbra, Portugal; carlosandrepires@gmail.com

**Keywords:** biochemical, reference values, Lusitano horse

## Abstract

To date, serum biochemical analytes reference intervals (RIs) in Lusitano horses have not been studied. This study aimed to establish the RIs for biochemical analytes following the American Society of Veterinary Clinical Pathology guidelines and to compare them with the general equine population’s RIs. Blood samples were collected from 76 clinically healthy adult Lusitano horses, and RIs of 22 biochemical variables were determined using Reference Value Advisor software. Lusitano horse-specific RIs are proposed for the following variables: total protein (3.9–7.0 g/dL), albumin (2.5–3.8 g/dL), globulin (1.1–3.7 g/dL), total bilirubin (1.0–5.6 mg/dL), direct bilirubin (0.09–0.68 mg/dL), indirect bilirubin (0.7–5.2 mg/dL), urea (21.0–38.9 mg/dL), creatinine (0.9–2.0 mg/dL), aspartate aminotransferase (150.7–345.1 IU/L), alkaline phosphatase (60.7–227.4 IU/L), lactate dehydrogenase (247.6–959.0 IU/L), glucose (75.5–131.5 mg/dL), cholesterol (58.6–125.2 mg/dL), sodium (129.0–154.9 mmol/L), phosphorus (1.8–4.5 mmol/L), chloride (90.3–107.0 mmol/L), and calcium (8.9–12.6 mg/dL). Different RIs were identified for healthy adult Lusitano horses for 17/22 serum biochemical analytes tested, emphasizing the need for breed-specific RIs to prevent misinterpretation of laboratory results.

## Simple Summary

Biochemical profiles are a relevant diagnostic tool during a horse’s routine or specific examinations. The Lusitano horse is a Portuguese breed that has gained worldwide acceptance in recent years for its athletic potential. However, the breed’s unique characteristics can influence the biochemical analytes, making it essential to establish reference intervals for accurate evaluations. Currently, there are no established reference intervals for the Lusitano horse, and this study aims to bridge this gap. This study involved 76 clinically healthy adult Lusitano horses, and their biochemical analytes were determined using an automated chemistry analyzer. Reference intervals (RIs) for 22 biochemical analytes were obtained. Lusitano horse-specific RIs were proposed for the following variables: total protein, albumin, globulin, total bilirubin, direct bilirubin, indirect bilirubin, urea, creatinine, aspartate aminotransferase, alkaline phosphatase, lactate dehydrogenase, glucose, cholesterol, sodium, phosphorus, chloride, and calcium. This study provides RIs for serum biochemical analytes in healthy adult Lusitano horses and emphasizes the need for breed-specific reference intervals to prevent misinterpretation of laboratory results.

## 1. Introduction

The Lusitano horse is one of the world’s most ancient breeds. It originates from Portugal but is reared worldwide, primarily in Brazil, France, and Spain. It is known for its great functionality and versatility, making it an important part of the Portuguese economy, with a registered population of about 5000 mares and 1000 stallions. In Portugal, equestrian sport and horse riding have been recognized as significant leisure time activities. Different varieties of horses are raised in the country; among them, the Lusitano breed is one of the foremost [1,2].

Clinical laboratory reference intervals (RIs) are important in clinical practice because clinicians must rely on them for the correct interpretation of laboratory results [3]. Biochemical profiles are used routinely by equine veterinarians to provide significant information about the specific changes in an organ or body system, the general response of the individual to some physiological or pathological conditions, and the metabolic state of horses. In addition, biochemical parameters can be good indicators of the response to treatment, the severity of a disease, as well as a horse’s well-being, health, and fitness levels. Most of the time, the absence of appropriate in-house RIs leads clinicians to use textbook RIs for their interpretation. In some cases, the interpretation of the results may be a challenge, and diagnostic errors can occur because most equine clinical textbooks containing RIs do not include descriptions of the animals to which they are applicable, conditions under which samples were obtained, the laboratory instruments used, or the specific statistical descriptors used. Additionally, biochemical RIs can be influenced by factors related to the animal, including age, sex, reproductive status, physical activity, breed, circadian rhythms, genetics, diet, environmental factors, and factors related to sampling or analytical techniques [3,4]. For the reasons cited above and because of the Lusitano horse’s worldwide popularity, it is important to use species-specific RIs to improve the accuracy of clinical decision making and prevent misdiagnosis.

Various studies on biochemical RIs in horses have been published reporting breed or type-specific RI in horses namely in Light horses [5], Clydesdale horses [6], Pakistan horses [7], Polish Konik horse [8], Iceland horses in Austria [9], Kiso horses [10], Friesian horses from North America [11] and Hucul horses [12]. However, to the best of our knowledge, there are currently no studies that have been conducted to establish specific biochemical RIs for the Lusitano horse. RIs specific to the Lusitano horses will help establish a physiologic database and should minimize errors in clinical decision making. Therefore, this study aimed to establish biochemical RIs for healthy adult Lusitano horses. In addition, we investigated whether RIs used for the general adult horse population could be applied to Lusitano horses.

## 2. Materials and Methods

### 2.1. Animal Selection Criteria

This study followed the guidelines published by the Clinical and Laboratory Standards Institute (CLSI) and the American Society of Veterinary Clinical Pathology (ASVCP) for establishing RIs in veterinary species [3,13]. All owners gave their informed consent for the use of their animals’ data. Procedures were carried out following the standards recommended by the Portuguese legislation for the protection of animals (Law no. 6 January 2022) and approved by the Animal Welfare Organism of the University of Trás-os-Montes e Alto Douro (UTAD)-Portugal (ORBEA) (project number 3441-e-DCV-2023 ORBEA).

The reference population included 76 client-owned healthy adult purebred Lusitano horses of both sexes, including 11 females and 65 males (58 intact and 7 geldings), aged 3–23 years (Mean = 9.6) from diverse owners abroad in Portugal and different stables throughout the country. The horses were kept under natural photoperiod and ambient temperature (climate classification: warm temperate with dry summers) in individual stalls, with free access to water, and were fed twice a day with hay (2% body weight) and commercial feed (14% protein and 4.35% fat). Almost all horses were for leisure, with five sport animals in the dressage modality, but not competing. Leisure horses are recreational animals, including riding school animals, countryside horse riding, and animals exercised in the arena for an average of 45 min, 4–5 times per week. Inclusion and exclusion criteria were established before sample collection. Adults were defined as horses with ages equal to or superior to three years old. Each owner was provided with a questionnaire for recording the horses’ use, husbandry, and pertinent medical history. Health was defined as no evidence of disease on the day of or in the 30 days preceding and following blood collection. A complete physical examination was performed by a veterinarian at the time of blood collection to establish the absence of disease. Horses were excluded if they were deemed unhealthy, had been vaccinated in the preceding 30 days, received medications other than routine anti-parasiticides, or were pregnant or lactating mares. Horses that displayed excitement or agitation at the time of sample collection, or had undergone strenuous exercise in the preceding 48 h, were also excluded. Finally, horses were excluded if subsequent examination of blood results (hematological or biochemical) led to a suspicion of underlying disease.

### 2.2. Sample Collection

Samples were obtained for 11 months, from November 2023 to October 2024. Horses were fasted for eight hours before blood collection. Samples were collected before the morning meal by jugular venepuncture using 20G blood collection needles and immediately transferred to a 10 mL sterile gel tube with no additive. Blood samples were allowed to clot for 30 min before centrifugation. Each gel tube was centrifuged at 1500× *g* for 5 min, and serum was aspirated, immediately frozen, and stored at −20 °C until analyzed. The serum samples were excluded if there was greater than mild hemolysis, icterus, or lipemia. One ml of blood was placed into a K3-ethylenediaminetetraacetic acid tube for routine hematology to assess the horses’ health status.

### 2.3. Biochemical Analyses

Biochemical analyses were performed on an automated chemistry analyzer (Daytona^®^ RX, Randox Laboratories, Crumlin, Northern Ireland) and the following analytes were reported: concentrations of albumin (ALB), total protein (TP), total bilirubin (TBIL), direct bilirubin (DBIL), bile acids (BA), urea, creatinine (CREA), cholesterol, (CHOL), triglycerides (TRIG), glucose (GLU), calcium (Ca), phosphorus (PHOS), chloride (Cl), potassium (K), and sodium (Na), and alkaline phosphatase (ALP), aspartate aminotransferase (AST), creatine kinase (CK), gamma-glutamyl transferase (GGT), and lactate dehydrogenase (LDH) activities. The following variables were calculated: globulin (GLOB = TP − ALB) concentration and indirect bilirubin (INBIL = TBIL − DBIL) concentration. Before each sample run, the analyzer was calibrated according to the manufacturer’s instructions, and control samples were analyzed.

### 2.4. Statistical Analysis

The descriptive analysis and calculations of the Reference Intervals (RIs) for the biochemical parameters were performed using the Reference Value Advisor V 2.1 software. It is a macro designed to calculate RIs with Microsoft Excel, available at http://www.biostat.envt.fr/reference-value-advisor/, accessed on 12 December 2024 [14].

The normality of the data was assessed using the Anderson–Darling test, and the presence of outliers was evaluated using Tukey’s method, both implemented in the Reference Value Advisor. The estimation of RI followed ASVCP guidelines [3], particularly regarding sample size (between 40 and 140 cases). For the estimation of the 95% RI, the parametric or robust method was used, with or without Box–Cox transformation, depending on the data distribution. RIs were considered validated if up to 10% of the values fell outside the published RIs. RIs were rejected if more than 10% of the values fell outside the published RIs. When published RIs were not validated, new RIs were suggested, following the Clinical and Laboratory Standards Institute (CLSI) and the American Society of Veterinary Clinical Pathology guidelines [3,13].

The Pearson Correlation Coefficient was used to study the correlation between variables. Correlations were considered weak if |R| < 0.25, moderate for 0.25 ≤ |R| < 0.50, strong for 0.50 ≤ |R| < 0.75, and very strong if |R| ≥ 0.75 [15]. The significance of differences between age groups was analyzed using Student’s *t*-test. These analyses were performed using IBM SPSS software, version 26 for Windows. A significance level of 5% (*p* < 0.05) was considered.

## 3. Results

The samples were composed of 76 clinically healthy Lusitano horses (11 females and 65 males) with a mean age of 9.6 years (age range, 3–23 years).

Results for biochemical data from Lusitano horses (mean, median, SD, minimum, maximum, proposed RIs for each parameter, lower and upper reference limit 90% CI) are summarized in Table 1.

Biochemical data from the Lusitano horses were compared with RIs for the general adult equine population on serum samples reported in one textbook [16]. The percentage of the Lusitano horse population that was outside non-breed-specific RIs and the Lusitano horse-specific RIs are reported in Table 2. Based on comparison with published RIs for the general adult equine population, new biochemical RIs were proposed for Lusitano horses for TP, ALB, GLOB, TBIL, DBIL, INBIL, urea, CREA, AST, ALP, LDH, GLU, CHOL, Na, PHOS, Cl, and Ca.

The Lusitano horses were divided into two age groups: 3–5 (n = 23, mean = 3.8, SD = 0.7) and ≥6 (n = 53, mean = 12.1, SD = 4.6, maximum = 23) years old. The results for biochemical data from each group (mean, median, SD, minimum, maximum, proposed RIs for each parameter, lower and upper reference limit 90% CI) are summarized in Table 3 and Table 4, respectively.

The correlation between the biochemical parameters and age, as well as the comparison of mean values between the two age groups, are presented in Table 5. There is a weak positive correlation statistically significant between both TBIL (R = 0.239, *p* = 0.037) and INBIL (R = 0.232, *p* = 0.044) and age. A moderate negative statistically significant correlation is observed between urea (R = −0.287, *p* = 0.012), AST (R = −0.349, *p* = 0.002), ALP (R = −0.343, *p* = 0.003), LHD (R = −0.279, *p* = 0.015), GLU (R = −0.260, *p* = 0.023), PHOS (R = −0.493, *p* = 0.000), and age. Some biochemical parameters, such as DBIL (*p* = 0.038)**,** CK (*p* = 0.005)**,** ALP (*p* = 0.038), LDH (*p* = 0.003), and PHOS (*p* = 0.000), show statistically significant differences between the two age groups.

## 4. Discussion

Breed-related differences in biochemical parameters may have an impact on clinical decisions. Although biochemical RIs for the general horse population have been used in clinical practice, these multibreed prediction intervals are influenced by the breed and somatotype [3]. Thus, the use of breed-specific biochemical RIs is ideal for the appropriate interpretation of the results of certain diagnostic tests. In this context, we have established specific biochemical RIs for Lusitano horses that provide a major tool in the interpretation of laboratory results, which will help prevent misinterpretation of laboratory results in clinical practice.

In this study, RIs were determined and compared with established non-breed-specific adult equine RIs according to the American Society of Veterinary Clinical Pathology guidelines [3] using Reference Value Advisor V 2.1 software. Different RIs were identified for Lusitano horses for 17/22 serum biochemical parameters tested, including TP, ALB, GLOB, TBIL, DBIL, INBIL, urea, CREA, AST, ALP, LDH, GLU, CHOL, Na, PHOS, Cl, and Ca when compared with RIs for the general adult equine population in one textbook [16]. For TP, GLOB, CREA, AST, ALP, CHOL, PHOS, Cl, and Ca, the new RIs were wider, while for ALB, TBIL, DBIL, INBIL, urea, LDH, GLU, and Na were higher. In this textbook, there is limited information, namely regarding the breed, the methodology of instruments used, and the methods of calculation of the RIs. Also, this can be true for many RIs used by veterinarians. One common source of variation in clinicopathologic data is the difference in results obtained from different laboratories [16]. Therefore, it is crucial to use validated RIs established by the specific laboratory to which samples are submitted. However, establishing breed-specific RIs for all horse breeds is challenging for laboratories, and as a result, these RIs are rarely breed-specific. This lack of information highlights the importance of using these IRs with caution in clinical practice and the need for RIs breed-specific. Thus, the breed-specific RIs found in this study provide a useful tool for the clinical interpretation of biochemical profiles in Lusitano horses.

Some studies report biochemical breed RIs in horses. However, older studies use obsolete analyzers [5,6], and in other studies [7,8,9,10,11,12] the employed methodology, analyzers, and interval calculation methods are different, so comparisons with this study are difficult. Some of the differences in our reported RIs are likely breed-related differences and highlight the need for new RIs in Lusitano horses. In our sample population, care was taken to avoid excitement during collection and recent strenuous exercise, and samples with moderate to marked hemolysis, icterus, or lipemia were excluded. Furthermore, the studied Lusitano horses were carefully screened for disease with a thorough history, physical examination, and hematological analyses; none were receiving medication. Therefore, it is unlikely that the biochemical parameters that were outside the published equine RIs can be related to the handling of the horses before sampling, preanalytical artifacts, muscular injury, or hepatobiliary disease. In Lusitano horses, the concentrations of TB and INBIL are higher than in published RIs. This trend was also reported in Polish Konik horses and can be explained by fasting hyperbilirubinemia [17] related to longer intervals between feedings. Fasting decreases the efficiency of plasma bilirubin removal in all species, but horses show a greater rise in plasma bilirubin [18]. High glucose concentrations could be caused by the stress associated with sampling, presumably from epinephrine or cortisol secretion, or also be caused by intense physical activities [19]. However, no other signs of stress (e.g., lymphopenia) were found in hematological analyses, and strenuous exercise in the preceding 48 h was an exclusion criterion in this study. Furthermore, Lusitano horses were fasted for eight hours before blood collection.

The relationship between blood biochemical parameters and training has been reported [20,21,22]. In horses, the most common blood indicators of health and fatigue states of muscle cells are CK, AST, and LDH activities [16,23]. These enzymes increased with exercise, which could be related to the muscular impact caused by a great effort [23]. The highest rate of muscle fatigue is observed during an endurance type of work [22]. Maśko et al. [22] observed that AST activity increased only in endurance horses because it requires more aerobic energy generation. In our study, Lusitano horses have higher LDH activities compared to IRs for the general horse population. However, CK activities were similar, and AST activities were lower. In addition, Lusitano horses were submitted to regular maintenance exercises. Therefore, it is unlikely that muscle fatigue is the cause of higher LDH activity. Metabolic adaptations to exercise in horses vary according to breed, training intensity, and specific sport modality, reflecting the changes in the functions of different systems and the type of energy utilized [24]. Lusitano horses, traditionally selected for classical dressage and working equitation [1,2], are typically subjected to lower aerobic demands compared to breeds used in endurance or racing. These differences influence physiological responses to exercise, particularly in terms of energy metabolism, muscle fiber composition, and oxidative stress, which could partially explain some differences in certain biochemical parameters observed in this study.

According to our results, TBIL and INBIL increased with age, and urea, AST, ALP, LDH, GLU, and PHOS decreased with age. Despite the increase in TBIL and INBIL with age, clinical signs potentially associated with hepatobiliary disease (icterus, weight loss, and ascites) [18] or hemolytic anemia (mucous membrane pallor, fatigue, depression, anorexia, and icterus) [25] were never detected in this study. Furthermore, other indicators of liver disease, such as AST, ALP, and LDH activities, decreased with age, which makes it unlikely that the existence of liver disease in older animals could explain these results. Standard biochemical indices of hepatocellular disease in horses include AST and LDH. ALP indicates both hepatocellular and biliary origin. However, these last three enzymes are not specific to the equine liver. ALP reflects biliary injury but is not specific to the liver, as it is also produced in the intestine, macrophages, and bone [26]. Thus, in this study, the decrease in ALP with age can be explained by the decrease in bone turnover. These findings suggest that age significantly influences various biochemical parameters that may be physiological and metabolic features of Lusitano horses, reinforcing the importance of considering age range in the clinical and laboratory evaluation of individuals.

Our sample population is a representative sample of the population of adult Lusitano horses and enabled the use of the methods for establishing RIs recommended [3]. However, since pregnant or lactating mares were not included, there is a disproportion between males and females, with a predominance of males, which is a limitation of the study. Andriichuk and Tkachenko (2017) [21] did not observe a significant difference in AST, ALT, and LDH activities between mares and stallions of Holsteiner horses. However, the authors of this study only compared AST, ALT, and LDH biochemical parameters. In another study, Thielebein et al. [27] identified sex-related differences in the serum levels of TP, ALB, GLOB, TRIG, Ca, selenium, ALP, AST, and GGT in Konik horses, with males exhibiting higher concentrations than females. In this study, differences in ALP, AST, GGT, CA, and selenium between males and females were observed only in stallions, suggesting that the influence of androgens could explain these variations. Therefore, further studies are needed to evaluate the effect of sex on biochemical parameters in horses and sex-specific RI for Lusitano horses.

## 5. Conclusions

This study suggests that for Lusitano horses, breed-specific biochemical parameters must be used to reduce the misinterpretation of laboratory results, since the RIs for the large majority of biochemical parameters are different from the general adult equine population. It is difficult to determine whether these differences are attributable to fixed breed-specific factors or acquired factors through environment, diet, and training. These RIs should provide a reliable baseline for adult Lusitano horses when samples are analyzed using the same methods and analyzers.

## Figures and Tables

**Table 1:** Biochemical reference intervals for adult Lusitano horses (n = 76).

Parameters	Mean	Median	SD	Min	Max	Reference Interval	Lower Reference Limit 90% CI	Upper Reference Limit 90% CI
Total protein (g/dL)	5.7	5.9	0.8	3.8	7.1	3.9–7.0	3.8–4.0	6.6–7.1
Albumin (g/dL)	3.3	3.3	0.3	2.4	3.9	2.5–3.8	2.4–2.7	3.8–3.9
Globulins (g/dL)	2.4	2.5	0.6	1.0	3.8	1.1–3.7	1.0–1.3	3.4–3.8
Bile acids (µmol/L)	7.0	6.3	3.4	1.2	18.2	2.3–15.8	1.2–2.8	13.9–18.2
Total bilirubin (mg/dL)	2.6	2.3	1.1	1.0	5.6	1.0–5.6	1.0–1.2	4.7–5.6
Direct bilirubin (mg/dL)	0.42	0.42	0.11	0.00	0.69	0.09–0.68	0.00–0.28	0.58–0.69
Indirect bilirubin (mg/dL)	2.2	1.9	1.1	0.6	5.3	0.7–5.2	0.6–0.8	4.0–5.3
Urea (mg/dL)	28.9	28.6	4.7	19.0	43.1	21.0–38.9	19.0–22.1	37.5–43.1
Creatinine (mg/dL)	1.4	1.5	0.2	0.9	2.0	0.9–2.0	0.9–1.0	1.8–2.0
CK (U/L)	180.4	172.0	55.1	96.0	330.0	100.6–315.2	96.0–107.6	299.0–330.0
AST (U/L)	241.1	241.5	49.6	147.0	346.0	150.7–345.1	147.0–159.4	325.6–346.0
ALP (U/L)	113.4	104.0	37.4	58.0	258.0	60.7–227.4	58.0–67.0	181.9–258.0
GGT (U/L)	13.2	12.7	5.2	1.3	30.3	1.5–26.9	1.3–6.0	21.0–30.3
LDH (U/L)	498.9	440.5	177.6	230.0	1131.0	247.6–959.0	230.0–304.9	860.6–1131.0
Glucose (mg/dL)	100.1	98.4	14.7	70.5	134.4	75.5–131.5	70.5–81.4	127.4–134.4
Triglycerides (mg/dL)	22.8	21.6	8.0	10.3	51.6	10.5–38.6	10.3–11.7	34.4–51.6
Cholesterol (mg/dL)	90.5	90.3	14.3	57.8	126.1	58.6–125.2	57.8–66.1	113.1–126.1
Sodium (mmol/L)	140.8	141.2	6.4	127.3	155.6	129.0–154.9	127.3–130.9	150.4–155.6
Phosphorus (mmol/L)	3.1	3.2	0.7	1.4	4.6	1.8–4.5	1.4–2.1	4.4–4.6
Potassium (mmol/L)	3.7	3.7	0.6	2.0	5.0	2.4–4.9	2.0–2.7	4.5–5.0
Chlorides (mmol/L)	98.0	98.3	4.0	90.3	108.7	90.3–107.0	90.3–92.0	104.9–108.7
Calcium (mg/dL)	11.1	11.3	1.0	8.5	13.4	8.9–12.6	8.5–9.2	12.3–13.4

ALP, alkaline phosphatase; AST, aspartate aminotransferase; CK, creatine kinase; GGT, gamma-glutamyl transferase; LDH, lactate dehydrogenase.

**Table 2:** Published reference intervals (RIs) and the new adult Lusitano horse-specific RIs.

Parameters	General Adult Horses RIs *	Outside RI (Below RI)	Within Published RI	Outside RI (Above RI)	Lusitano Horses RI
Total protein (g/dL)	5.2–7.9	18.4%	81.6%		**3.9–7.0**
Albumin (g/dL)	2.6–3.7	3.9%	89.5%	6.6%	**2.5–3.8**
Globulins (g/dL)	2.62–4.04	64.5%	35.5%		**1.1–3.7**
Total bilirubin (mg/dL)	1–2		31.6%	68.4%	**1.0–5.6**
Direct bilirubin (mg/dL)	0–0.4		38.2%	61.8%	**0.09–0.68**
Indirect bilirubin (mg/dL)	0.2–2		52.6%	47.4%	**0.7–5.2**
Urea (mg/dL)	10–24		18.4%	81.6%	**21.0–38.9**
Creatinine (mg/dL)	1.2–1.9	14.5%	82.9%	2.6%	**0.9–2.0**
CK (IU/L)	108–430	5.3%	94.7%		100.6–315.2
AST (IU/L)	226–366	42.1	57.9%		**150.7–345.1**
ALP (IU/L)	143–395	84.0%	16.0%		**60.7–227.4**
GGT (IU/L)	7–54	9.3%	90.7%		1.5–26.9
LDH (IU/L)	162–412		38.2%	61.8%	**247.6–959.0**
Glucose (mg/dL)	75–115	1.3%	85.5%	13.2%	**75.5–131.5**
Cholesterol (mg/dL)	75–150	13.2%	86.8%		**58.6–125.2**
Sodium (mmol/L)	132–146	6.6%	69.7%	23.7%	**129.0–154.9**
Phosphorus (mmol/L)	3.1–5.6	46.7%	53.3%		**1.8–4.5**
Potassium (mmol/L)	2.4–4.7	1.3%	96.1%	2.6%	2.4–4.9
Chlorides (mmol/L)	99–109	61.8%	38.2%		**90.3–107.0**
Calcium (mg/dL)	11.2–13.6	42.7%	57.3%		**8.9–12.6**

ALP, alkaline phosphatase; AST, aspartate aminotransferase; CK, creatine kinase; GGT, gamma-glutamyl transferase; LDH, lactate dehydrogenase. RIs were considered validated if ≤ 10% of the Lusitano horse’s values fell outside the published RIs. RIs were rejected if >10% of the values fell outside the published RIs. When published RIs were not validated, new RIs were suggested and reported in bold, following the Clinical and Laboratory Standards Institute (CLSI) and the American Society of Veterinary Clinical Pathology guidelines [3,13]. * Stămpfli and Oliver-Espinosa [16]. * Without reference intervals for bile acids and triglycerides.

**Table 3:** Biochemical reference intervals for Lusitano horses with 3–5 years (n = 23).

Parameters	Mean	Median	SD	Min	Max	Reference Interval	Lower Reference Limit 90% CI	Upper Reference Limit 90% CI
Total protein (g/dL)	5.6	5.9	0.9	3.8	7.0	3.8–8.0	3.0–4.5	7.3–8.3
Albumin (g/dL)	3.3	3.3	0.4	2.5	3.8	2.2–3.9	1.7–2.7	3.8–4.0
Globulins (g/dL)	2.3	2.4	0.7	1.0	3.4	1.0–3.8	0.5–1.5	3.4–4.1
Bile acids (µmol/L)	6.2	5.6	2.2	2.7	10.5	2.5–12.4	2.0–3.2	9.7–14.8
Total bilirubin (mg/dL)	2.2	2.1	0.9	1.0	3.8	0.8–4.5	0.7–1.1	3.6–5.3
Direct bilirubin(mg/dL)	0.39	0.41	0.11	0.10	0.62	0.12–0.60	0.00–0.24	0.54–0.66
Indirect bilirubin (mg/dL)	1.8	1.6	0.9	0.6	3.3	0.5–4.1	0.4–0.7	3.2–4.8
Urea (mg/dL)	28.7	28.2	3.2	22.5	37.9	22.7–36.5	21.6–24.6	33.5–39.6
Creatinine (mg/dL)	1.4	1.4	0.3	0.9	2.0	0.9–2.0	0.8–1.1	1.8–2.2
CK (U/L)	207.0	210.0	57.3	108.0	330.0	104.0–344.4	82.8–127.8	298.2–392.8
AST (U/L)	255.7	259.0	50.9	169.0	335.0	138.0–358.4	100.7–185.0	334.5–385.3
ALP (U/L)	137.7	125.0	47.7	68.0	258.0	66.4–278.1	56.3–78.6	213.7–339.4
GGT (U/L)	11.6	11.3	4.5	1.3	21.8	1.9–20.8	0.8–5.0	17.2–24.0
LDH (U/L)	587.8	519.0	197.0	353.0	945.0	284.6–1209.7	232.0–328.0	881.1–1580.1
Glucose (mg/dL)	103.9	100.5	16.2	81.7	134.4	77.9–150.0	73.9–82.4	128.8–173.9
Triglycerides (mg/dL)	23.2	22.1	7.0	11.6	37.5	8.9–38.9	5.8–12.0	34.1–43.6
Cholesterol (mg/dL)	91.1	92.1	15.9	57.8	115.1	49.0–120.0	32.9–64.6	112.4–126.2
Sodium (mmol/L)	141.4	140.5	5.9	132.6	155.6	128.4–153.6	124.9–132.0	149.1–157.0
Phosphorus (mmol/L)	3.5	3.4	0.6	2.8	4.6	2.6–5.4	2.5–2.8	4.4–6.4
Potassium (mmol/L)	3.9	4.0	0.6	2.4	5.0	2.4–5.1	1.7–2.9	4.8–5.3
Chlorides (mmol/L)	98.3	98.5	4.1	90.3	108.7	90.6–108.4	88.7–93.1	104.9–111.6
Calcium (mg/dL)	11.2	11.6	0.9	9.2	12.4	9.6–13.8	8.6–10.3	13.1–14.1

ALP, alkaline phosphatase; AST, aspartate aminotransferase; CK, creatine kinase; GGT, gamma-glutamyl transferase; LDH, lactate dehydrogenase.

**Table 4:** Biochemical reference intervals for Lusitano horses with ≥ 6 years (n = 53).

Parameters	Mean	Median	SD	Min	Max	Reference Interval	Lower Reference Limit 90% CI	Upper Reference Limit 90% CI
Total protein (g/dL)	5.8	5.9	0.6	4.1	7.1	4.2–7.0	4.1–4.5	6.6–7.1
Albumin (g/dL)	3.2	3.3	0.3	2.4	3.9	2.5–3.9	2.4–2.7	3.8–3.9
Globulins (g/dL)	2.5	2.5	0.6	1.1	3.8	1.1–3.8	1.1–1.6	3.4–3.8
Bile acids (µmol/L)	7.1	6.6	3.3	2.4	15.5	2.5–15.4	2.4–3.1	13.5–15.5
Total bilirubin (mg/dL)	2.7	2.6	1.1	1.0	5.6	1.0–5.6	1.0–1.3	4.7–5.6
Direct bilirubin (mg/dL)	0.44	0.43	0.09	0.23	0.69	0.26–0.69	0.23–0.32	0.57–0.69
Indirect bilirubin (mg/dL)	2.3	2.1	1.1	0.7	5.3	0.7–5.3	0.7–0.8	4.0–5.3
Urea (mg/dL)	29.0	29.1	5.3	19.0	43.1	19.8–41.5	19.0–21.8	37.4–43.1
Creatinine (mg/dL)	1.5	1.5	0.2	0.9	2.0	0.9–2.0	0.9–1.1	1.8–2.0
CK (U/L)	168.9	162.0	50.4	96.0	313.0	97.8–310.2	96.0–105.0	272.9–313.0
AST (U/L)	234.8	230.0	48.2	147.0	346.0	148.4–345.7	147.0–158.9	317.0–346.0
ALP (U/L)	102.7	98.5	25.7	58.0	177.0	59.0–168.2	58.0–65.7	147.1–177.0
GGT (U/L)	13.9	13.6	5.3	1.6	30.3	2.9–29.0	1.6–6.2	21.9–30.3
LDH (U/L)	450.1	424.0	127.9	230.0	906.0	236.7–835.0	230.0–302.0	663.2–906.0
Glucose (mg/dL)	98.5	98.1	13.8	70.5	129.9	72.4–129.0	70.5–78.3	121.3–129.9
Triglycerides (mg/dL)	22.1	19.9	7.5	10.3	37.1	10.4–36.5	10.3–11.3	34.2–37.1
Cholesterol (mg/dL)	89.6	89.8	13.0	58.7	126.1	61.0–121.3	58.7–68.4	108.6–126.1
Sodium (mmol/L)	140.6	141.8	6.7	127.3	154.9	128.0–154.5	127.3–130.6	151.3–154.9
Phosphorus (mmol/L)	2.9	2.9	0.6	1.4	4.4	1.6–4.3	1.4–2.1	4.0–4.4
Potassium (mmol/L)	3.6	3.7	0.5	2.0	4.7	2.2–4.6	2.0–2.7	4.4–4.7
Chlorides (mmol/L)	97.8	98.2	3.9	90.3	106.9	90.7–106.2	90.3–92.1	104.1–106.9
Calcium (mg/dL)	11.0	11.3	1.0	8.5	13.4	8.7–13.1	8.5–9.1	12.2–13.4

ALP, alkaline phosphatase; AST, aspartate aminotransferase; CK, creatine kinase; GGT, gamma-glutamyl transferase; LDH, lactate dehydrogenase.

**Table 5:** Correlation between biochemical parameters and age, and comparison of mean values between the two age groups.

Correlations			3–5 Years (n = 23)		≥6 Years (n = 53)		*p*
Parameters	R	*p*	M	SD	M	SD	
Total protein (g/dL)	0.154	0.184	5.60	0.94	5.75	0.70	0.492
Albumin (g/dL)	−0.043	0.712	3.29	0.35	3.24	0.33	0.581
Globulins (g/dL)	0.217	0.059	2.31	0.66	2.51	0.59	0.201
Bile acids (µmol/L)	−0.100	0.392	6.82	3.79	7.08	3.27	0.763
Total bilirubin (mg/dL)	0.239	0.037	2.32	1.05	2.71	1.12	0.161
Direct bilirubin (mg/dL)	0.110	0.344	0.38	0.14	0.44	0.09	0.038
Indirect bilirubin (mg/dL)	0.232	0.044	1.94	1.00	2.28	1.12	0.218
Urea (mg/dL)	−0.287	0.012	28.71	3.24	29.02	5.25	0.759
Creatinine (mg/dL)	0.012	0.915	1.39	0.25	1.48	0.22	0.118
CK (U/L)	−0.212	0.066	207.04	57.26	168.91	50.43	0.005
AST (U/L)	−0.349	0.002	255.74	50.87	234.75	48.21	0.091
ALP (U/L)	−0.343	0.003	137.74	47.70	102.65	25.72	0.003
GGT (U/L)	0.061	0.600	11.58	4.46	13.91	5.32	0.071
LDH (U/L)	−0.279	0.015	611.39	223.35	450.09	127.93	0.003
Glucose (mg/dL)	−0.260	0.023	103.90	16.25	98.50	13.79	0.142
Triglycerides (mg/dL)	0.089	0.446	23.16	7.03	22.62	8.47	0.791
Cholesterol (mg/dL)	0.068	0.558	92.54	17.06	89.63	13.04	0.419
Sodium (mmol/L)	−0.203	0.078	141.43	5.86	140.55	6.68	0.587
Phosphorus (mmol/L)	−0.493	0.000	3.54	0.58	2.94	0.63	0.000
Potassium (mmol/L)	−0.035	0.767	3.89	0.62	3.62	0.55	0.067
Chlorides (mmol/L)	0.052	0.654	98.26	4.13	97.82	3.93	0.663
Calcium (mg/dL)	−0.079	0.500	11.22	0.92	11.03	1.01	0.435

## Data Availability

No new data were created or analyzed in this study. The original contributions presented in this study are included in the article. Further inquiries can be directed to the corresponding author.
